# Recurrent and persistent refractive errors after restarting isotretinoin treatment: a case report

**DOI:** 10.3389/fphar.2026.1772498

**Published:** 2026-04-02

**Authors:** Ming-xing Gong, Shan-shan Li, He Huang, Fen Zhou

**Affiliations:** 1 Department of Pharmacy, Chongqing Red Cross Hospital, Chongqing, China; 2 Department of Clinical Laboratory, Chongqing Sanbo Changan Hospital, Chongqing, China; 3 Department of Dermatology, Chongqing Red Cross Hospital, Chongqing, China

**Keywords:** acne vulgaris, astigmatism, disease recurrency, isotretinoin, myopia, refractive error

## Abstract

**Background:**

Isotretinoin is a vitamin A derivative widely used for moderate-to-severe acne, and is known to cause multiple systemic and ocular adverse events. Refractive errors, such as myopia and astigmatism, are commonly considered reversible after discontinuation of isotretinoin treatment. However, the visual changes and subsequent prognosis after re-exposure to isotretinoin are rarely reported.

**Case Presentation:**

A 21-year-old male initiated oral isotretinoin at 10 mg twice daily for acne vulgaris. Twelve days later, the patient developed acute visual deterioration. The eye examination revealed mild myopic astigmatism in the right eye and mild myopia in the left eye. The patient’s visual acuity returned to normal at approximately 20 days after isotretinoin cessation. Then, the patient later self-restarted isotretinoin (10 mg, twice daily) for 10 days, which again resulted in decreased visual acuity. Although the patient discontinued isotretinoin, the visual acuity did not recover to baseline over a 14-month follow-up period. The Naranjo Adverse Drug Reaction Probability Scale was eight points, indicating a “probable” causal relationship between isotretinoin and refractive error.

**Conclusion:**

Myopia and astigmatism might happen concurrently after isotretinoin administration. In addition, recurrent and persistent refractive errors might occur upon restarting isotretinoin in patients with prior history of isotretinoin-related ocular complications. Clinicians should consult patients to take extra precautions on isotretinoin medication when they have a history of ocular disease or ocular complications with isotretinoin administration.

## Introduction

Isotretinoin is a retinoid approved by the U.S. Food and Drug Administration in 1982 for severe acne treatment, and exerts its therapeutic effects through suppressing sebaceous lipid synthesis, inhibiting *Cutibacterium acnes* proliferation, and modulating inflammatory and immune responses (Melnik, 2017). Although highly effective, isotretinoin is associated with a broad spectrum of adverse events, most of which resemble vitamin A toxicity. These adverse events range from common mucocutaneous issues, such as xeroderma, cheilitis, dry eyes, and blepharitis, to more severe complications, including hypertriglyceridemia, thrombocytopenia, inflammatory bowel disease, edema, suicidal ideation, and anaphylaxis. In addition, teratogenicity remains its most serious toxicity, necessitating strict contraception in women of childbearing potential.

Ocular complications are well-documented adverse events from isotretinoin administration. Affected patients can present with dry eyes, visual acuity changes, nyctalopia, photophobia, and diplopia (Lamberg et al., 2023; Tao et al., 2025). The majority of these ocular complications are reversible, with symptoms gradually resolving, and the patient’s eye vision returning to baseline after discontinuation of isotretinoin. However, there is limited research on its recurrent ocular adverse events and the subsequent prognosis after restarting isotretinoin. The present study reports a case of early-onset isotretinoin-induced refractive error, with concurrent myopia and astigmatism. In addition, the patient had recurrent and persistent visual impairment after re-exposure to isotretinoin. Previous literature was also further reviewed, with the purpose of reminding clinicians to pay attention to ocular complications when prescribing isotretinoin, especially in patients with prior ocular disease or complications from isotretinoin treatment.

## Case report

On 20 June 2024, a 21-year-old male presented to our dermatology clinic with a 1-year history of facial papules, pustules, and nodules that had worsened over the previous 2 months. The patient was diagnosed with moderate acne vulgaris, and prescribed oral isotretinoin at 10 mg twice daily, along with a one-time compound acid peel treatment (Mediderma type II, Mediderma, USA). On 2 July 2024, the patient reported eye discomfort with decreased vision. The patient denied any previous history of eye disease or similar symptoms. Isotretinoin was immediately discontinued, and the patient was referred to the ophthalmology department.

In the ophthalmology department, the examination revealed the following:

1. Visual acuity and refraction (visual acuity was measured using the decimal system, and refraction was measured using the combination of subjective refraction and objective refraction. The objective refraction was measured by an auto-refractometer, NIDEK Co. Ltd., Japan):

Right eye: uncorrected 0.6 (−0.50 DS/-0.50 DC ×130), corrected to 1.2.

Left eye: uncorrected 0.9 (−0.50 DS), corrected to 1.2.

2. Intraocular pressure: 13 mmHg (right), 14.5 mmHg (left)
3. Slit-lamp exam: clear corneas, clear anterior chambers, and transparent lenses
4. Ultrasonography: mild vitreous opacities
5. Fundus examination: normal retina and macula bilaterally
6. Optical coherence tomography: normal foveal and perifoveal morphology, no disc edema or cupping, and normal retinal nerve fiber layer thickness.

The examination results revealed no signs of glaucoma, macular pathology, or optic nerve abnormalities. The mild vitreous opacity lacked features indicative of an acute etiology, and was deemed incidental. The patient’s decreased vision was correctable, and both anterior and posterior segment structures were within normal limits. The patient was diagnosed with mild myopic astigmatism in the right eye and mild myopia in the left eye. Approximately 20 days following the cessation of isotretinoin therapy, the patient’s vision acuity returned to baseline levels.

During a telephone follow-up on 11 October 2024, the patient reported that he restarted isotretinoin (10 mg, twice daily) at home on 5 August 2024 due to persistent acne. After 10 days of therapy, the patient experienced blurry vision again. The patient discontinued the medication. Over a 14-month follow-up period until October 2025, the patient’s vision failed to recover to baseline levels. Unfortunately, the patient declined a follow-up ophthalmic evaluation despite multiple attempts to schedule an appointment for him.

The patient denied any past history of ocular disease and changes in lifestyle, visual habits, environmental exposures, concomitant medications, or systemic symptoms between the two episodes of visual impairment. The comprehensive ophthalmic evaluation during the first episode excluded organic ocular pathology. Given the clear temporal association, recurrence upon rechallenge, and absence of alternative explanations, isotretinoin was identified as the sole causative factor. The patient had a Naranjo Adverse Drug Reaction Probability Scale of eight points, indicating a “probable” causal relationship.

## Discussion

Refractive error refers to the inability of parallel light rays to focus precisely on the retina, leading to blurred vision. Common types of refractive error include myopia, hyperopia, and astigmatism (Wang et al., 2021; Schiefer et al., 2016). Myopia and hyperopia result from the mismatch between ocular axial length and refractive power, and are typically corrected with spherical lenses (Schiefer et al., 2016). Astigmatism involves two focal planes that may lie anterior to, posterior to, or across the retinal plane, depending on its coexistence with myopia or hyperopia (Harb and Wildsoet, 2019). Although refractive errors are largely attributed to genetic and environmental factors, drug-induced cases have been previously reported (Patasova et al., 2021; Williams and Hammond, 2025). Isotretinoin is one of the medications that can cause refractive errors. The majority of these refractive errors are reversible after isotretinoin discontinuation. We report this case to show that myopia and astigmatism can happen concurrently after isotretinoin administration. In addition, re-exposure to isotretinoin in patients with a history of isotretinoin-related ocular complications might lead to recurrent and persistent visual impairment. This has rarely been previously reported.

### Causality assessment

The patient had no pre-existing eye disease, and developed visual deterioration only after initiating isotretinoin. The patient’s visual acuity fully recovered after isotretinoin discontinuation, and deteriorated again on rechallenge, strongly suggesting a drug-related effect. In addition, the Naranjo Adverse Drug Reaction Probability Scale was eight points, indicating a “probable” causal relationship between isotretinoin and refractive errors. Therefore, the investigators considered that isotretinoin was responsible for the patient’s refractive errors.

### Potential mechanisms

Several mechanisms may explain the isotretinoin-induced refractive changes:

1. Ocular surface alteration-induced changes

Isotretinoin commonly causes dry eyes, which is characterized by tear-film instability and shortened tear break-up time. Tear-film abnormalities can lead to an irregular corneal surface, contributing to astigmatic changes or fluctuations in refractive power, ultimately causing visual blur and refractive error (Barros et al., 2025; Musolf et al., 2023). Since corneal parameters are the major determinant of refractive error (Musolf et al., 2023), ocular surface disease may act as an indirect mechanism. In the present case report, the patient did not report dry eyes, and the cornea was clear with no abnormalities on examination, suggesting that the refractive changes were due to functional effects on the visual pathway or signals related to refractive development, rather than being caused by ocular surface alteration.

2. Disruption of retinoic acid pathways in visual and refractive development

Retinoic acid signaling regulates visual function and refractive development. Retinoic acid derivatives have been shown to partially restore adult visual plasticity and modify primary visual cortex neuronal responses (Huh et al., 2022). All-trans retinoic acid plays a crucial role in ocular growth by regulating corneal homeostasis and axial elongation through NF-κB-retinoid pathways (Yu et al., 2021; Isl et al., 2022). As a vitamin A derivative, isotretinoin may theoretically perturb these pathways, altering refractive balance.

3. Neuromolecular factors linked to refractive error

Sensory-neural disturbances may interfere with accommodative function, indirectly leading to refractive abnormalities^7^. The *GJD2* gene, which encodes the connexin protein involved in ocular biometry and axial length regulation, has been implicated in myopia development (Quint et al., 2021). Although no direct evidence has linked isotretinoin to GJD2 pathways, the retinoid-mediated modulation of visual function suggests a plausible interaction.

### Comparisons with previous literature

Drug-induced refractive error related to isotretinoin is rare. A prospective study published in 2020 demonstrated significant increases in myopia and axial length at 6 months of isotretinoin therapy, independent of age, sex, dose, or baseline refractive state (Yasar et al., 2020) (Table 1).

**TABLE 1:** Previous literature and the present case report on isotretinoin-related eye illnesses.^a^

Author, year	Study design	Case number	Medical history	Ocular illnesses	Duration from isotretinoin to eye symptoms	Outcomes
Yasar et al. (2020)	Prospective study	Isotretinoin group 47 Control group 45	N/A	Myopia, increased axial length in isotretinoin group	Six months	N/A
Saraswat (2011)	Case report	A 22-year-old male	No	Myopia	Two months	No visual change after isotretinoin discontinuation
Park and Lee (2017)	Case report	A 28-year-old woman	No	Myopia with increased ocular pressure	Two weeks	Resolved after isotretinoin discontinuation
Qureshi et al. (2021)	Case report	A 29-year-old woman	Myopia	Myopia	Four weeks	No visual change after isotretinoin discontinuation
Current case	Case report	A 21-year-old man	No	Myopia with astigmatism	Twelve days	Resolved after isotretinoin discontinuation, but recurred after re-started it

^a^ All patients were prescribed isotretinoin due to acne.

The investigators identified several previous case reports that described isotretinoin-induced myopia or myopic progression, with no previously reported cases of astigmatism (Table 1). A 22-year-old Indian male developed progressive, irreversible bilateral high myopia after 2 months of isotretinoin therapy (Saraswat, 2011). A 28-year-old woman developed transient myopia after 2 weeks of isotretinoin use, with full recovery after medication discontinuation (Park and Lee, 2017). A 29-year-old woman with a history of myopia corrected by laser-assisted *in situ* keratomileusis experienced recurrent myopia at 4 weeks after taking isotretinoin, with no recovery after medication withdrawal (Qureshi et al., 2021). As far as we know, this was the first case report on concurrent myopia and astigmatism after isotretinoin administration. This is a reminder that several eye disorders might simultaneously happen due to side effects from medications. A comprehensive ophthalmic evaluation should be performed in patients with suspected eye complications from isotretinoin intake.

Most previously reported cases have revealed an onset after isotretinoin therapy initiation for more than 14 days. In contrast, the present patient developed symptoms at 12 days, which is earlier than those previously documented. Although short-term drug-induced changes might be reversible, re-exposure might lead to irreversible refractive errors, as illustrated by the present patient’s second episode of blurry vision.

The strength of the present study was that the study reported a patient with recurrent refractive errors after restarting isotretinoin, reminding clinicians to pay special attention to patients with prior history of complications from isotretinoin intake. Meanwhile, the present case report had several limitations. First, there were no patient baseline eye examination findings, since the patient had no prior history of eye disease, and never received a formal ophthalmologic examination. Second, follow-up ophthalmologic examination data were unavailable, because the patient lived in a rural area, and declined to travel a long distance for follow-up ophthalmologic examinations. In addition, information on other detailed ophthalmologic assessments, such as corneal measurements, Schirmer test, and Break-Up Time, were not obtained, which might identify other potential ocular conditions associated with isotretinoin intake. Despite these limitations, we consider that reporting the present case remains important to increase clinicians’ awareness of isotretinoin-related ocular complications. That is, clinicians should pay special attention to patients with a history of ocular disease when prescribing isotretinoin, and educate these patients not to take isotretinoin without prior consultation with a clinician. The pathophysiological mechanism underlying the relationship between isotretinoin and ocular disorders requires further studies.

## Conclusion

Isotretinoin might cause refractive error, presenting with concurrent myopia and astigmatism, which could be reversible with early detection and discontinuation of isotretinoin treatment. However, restarting isotretinoin might result in recurrent and persistent refractive errors. Clinicians should exercise heightened vigilance when managing patients with prior history of ocular disease, especially patients with ocular disease related to isotretinoin treatment. Patients with a history of ocular complications from isotretinoin intake should be consulted, in order to weigh the risks and benefits when administrating isotretinoin again.

## Data Availability

The raw data supporting the conclusions of this article will be made available by the authors, without undue reservation.
